# Development of a Virtual Collision Sensor for Industrial Robots

**DOI:** 10.3390/s17051148

**Published:** 2017-05-18

**Authors:** Marina Indri, Stefano Trapani, Ivan Lazzero

**Affiliations:** 1 Dipartimento di Automatica e Informatica, Politecnico di Torino, Corso Duca degli Abruzzi 24, Torino 10129, Italy; stefano.trapani@polito.it; 2 COMAU SpA, Via Rivalta 30, Grugliasco (TO) 10095, Italy; ivan.lazzero@comau.com

**Keywords:** collision detection, industrial manipulators, virtual sensors

## Abstract

Collision detection is a fundamental issue for the safety of a robotic cell. While several common methods require specific sensors or the knowledge of the robot dynamic model, the proposed solution is constituted by a virtual collision sensor for industrial manipulators, which requires as inputs only the motor currents measured by the standard sensors that equip a manipulator and the estimated currents provided by an internal dynamic model of the robot (i.e., the one used inside its controller), whose structure, parameters and accuracy are not known. The collision detection is achieved by comparing the absolute value of the current residue with a time-varying, positive-valued threshold function, including an estimate of the model error and a bias term, corresponding to the minimum collision torque to be detected. The value of such a term, defining the sensor sensitivity, can be simply imposed as constant, or automatically customized for a specific robotic application through a learning phase and a subsequent adaptation process, to achieve a more robust and faster collision detection, as well as the avoidance of any false collision warnings, even in case of slow variations of the robot behavior. Experimental results are provided to confirm the validity of the proposed solution, which is already adopted in some industrial scenarios.

## 1. Introduction

Collision detection and avoidance are fundamental issues for the safety of a robotic cell in any industrial environment, not only in the future when the presence of collaborative robots directly working with humans is expected to grow, but also in current, standard production lines. Errors in programming, as well as unexpected events or objects in the robot workspace may lead to collisions, despite the monitoring activity of collision avoidance procedures (e.g., like in [1,2]), if they are based on a detailed prior knowledge of the cell and of the elements in it. Using an advanced collision detection procedure, collisions can be quickly detected, so that an appropriate reaction, reducing their effects, can be planned and executed.

The matter has been widely investigated in the most recent years with particular application to the case of Human-Machine Interaction (HMI) [3,4], which is effectively and safely achieved through the adoption of particular strategies and technological solutions, like the usage of Light Weight Robots (LWRs). These robots are specifically designed for the interaction with unknown environments and humans, thanks to a light-weight mechanical design minimizing their inertia; they are also equipped with several integrated sensors and advanced control strategies for compliant manipulation [3]. The coverage of the manipulator with a visco-elastic material [5] is another effective strategy to mitigate the collision effects thanks to the property of the material and to detect contact forces. Specific solutions have been also adopted to reduce the contact forces in the case of collisions, decoupling the inertia of the link with that of the rotor. e.g., introducing an elastic transmission between the actuator and the link; other more advanced approaches use a double actuator or implement a Variable Stiffness Transmission (VST), like in [4].

It is evident that all of these approaches are devoted to HMI applications and are implementable for robots having a particular mechanical structure and, most of all, enhanced sensor equipment, which cannot be constituted by only the proprioceptive sensors that are present in the standard industrial robots, providing joint positions and velocities and motor currents: the presence of some kind of force/torque sensor is mandatory for such solutions.

In the industrial context, the collision detection problem is characterized by different goals, safety levels and sensor facilities. In this scenario, the main goal is the preservation of the robot mechanical parts in the case of impact, as well as the robustness of the procedure to false collision detection; the sensitivity of the collision detection method and the possibility of varying it are fundamental to avoid the wrong detection of collisions that never actually occurred, with the consequent unjustified stop of the production cycle.

A further important application of an effective collision detection procedure in the industrial context is aimed at monitoring the correct execution of the programmed task. Sometimes, a failure in the process may occur, causing various possible troubles in the cell; if the effects of the fault on the manipulator are similar to those caused by a collision, a collision detection procedure can detect the anomaly and then immediately stop the robot. A typical example is given by the case in which a spot welding gun electrode gets stuck on the work-piece at the end of the welding process.

Several methods can be found in the literature to detect collisions for industrial robots, but some of them are based again on the usage of sensors that are not included in the standard equipment of manipulators. For example, some techniques use vision sensors [6] to detect the presence of unmodeled objects in the cell, or exploit algorithmic approaches, typical of methods using proprioceptive sensors only, but making use also of extra sensors (e.g., torque sensors), to improve the performance of the procedure [7,8].

The most common methods using only proprioceptive sensors typically divide the collision detection problem into two sub-problems: the computation of a residual term depending from external forces and the comparison of such a term with a predefined threshold function. The residual term is generally determined as the difference between a signal measured by the available proprioceptive sensors, somehow related to the total joint torques, and the same physical quantity mathematically computed in absence of collisions. Most of the approaches using only proprioceptive sensors can be classified as model based [9,10], because they require the full knowledge of the robot dynamic model and of its parameters to compute the residual term, for which they often make use of observers (e.g., the residual observer in [9,11,12] or the disturbance observer in [10,13]). The main drawbacks of these methods, which will be analyzed in detail in SubSection 1.1, are related to the limited portability of their software implementation, since the dynamic model of the specific considered robot is required each time, and to the dependance of their performances on the quality of the used dynamic model. Adaptive solutions are sometimes adopted to cope with model error problems, increasing their complexity and the related computational burden in the case of real-time implementation.

Non-model-based approaches have been rarely investigated, even if they would be preferable in the industrial context, because their software implementation can be independent from the particular robot, thus achieving a high portability to different classes of manipulators, as well as to various industrial applications and processes. The most interesting non-model-based method to be mentioned is the grey-box approach proposed in [14]. The procedure relies on the knowledge of the general structure of model-errors and uses this information to re-map the model error into five terms, one of which includes the external force. The five terms are computed without using the parameters of the model, but through an on-line estimation process carried out by a recursive least square algorithm.

The solution proposed in this paper is constituted by a virtual collision sensor for industrial manipulators, which has been developed through a research activity carried on by the collaboration of Politecnico di Torino and COMAU SpA, and motivated by critical situations that occurred in some factories and production lines using COMAU manipulators, as well as by the goal of improving a previous COMAU software solution for collision detection, which required a high level of customization and long warm up phases. The core of the virtual collision sensor is given by the collision detection procedure that was proposed in [15], which is here revised and enhanced, introducing the possibility of automatically customizing the sensor sensitivity for a specific robotic application and, subsequently, slowly adapting it. This new functionality enhances the sensor ability of recognizing even light collisions while avoiding the risk of generating false collision alarms and allows coping with possible slow variations of the robot behavior. The computational burden is anyway kept low to meet the requirements for real-time implementation in the robot control software; moreover, thanks to such an enhanced sensitivity, a more robust and faster detection is achieved, when a collision actually occurs.

The inputs of the developed virtual collision sensor are the motor current values measured by the standard sensors mounted on the Direct Current (DC) motors actuating the joints and the corresponding estimated currents as provided by the original robot controller, on the basis of an internal dynamic model of the manipulator, whose structure and parameters are not available. In this sense, the proposed approach can be considered as a non model-based one, since the virtual sensor simply uses the same information about the robot dynamics employed within the robot controller, without any knowledge about how this information is obtained, i.e., without any knowledge of the quality of the adopted dynamic model inside the controller. The only actual assumption, that the proposed approach relies on is that some estimate of the motor currents is available, as it always happens in the controller of any industrial manipulator, independently of the specific control scheme. The model error is directly estimated within the virtual sensor, taking into account the robot behavior in its various motion phases through the analysis of the current trends. A smart threshold function is thus generated, to be compared with the current residue to detect collisions. Finite State Machines (FSMs) are used to manage the computation of the threshold function and its comparison with the current residue only in the phases in which collisions can actually occur, so to enhance the efficiency of the procedure, as well as to handle the learning and adaptation process of the sensor sensitivity. This last new functionality is applicable to cyclic motion processes and is enabled only if and when the user requests it. It is based on a learning phase, carried out in parallel with the standard working of the virtual collision sensor, during which the current residue is monitored to determine, for the specific robot, the “best” value of the current parameter, associated with the minimum collision torque that the virtual sensor should be able to detect on each joint. The idea of monitoring the robot behavior during some cycles is common to various approaches for anomaly detection (see, e.g., [16] and the references therein), but in our case, there is no model to be trained (as in any proper learning process), but simply the continuous update of the parameter defining the sensor sensitivity on each joint, in order to avoid the risk of false collisions. In [17], a sensorless tool collision detection method is proposed for multi-axis machine tools, based on the information acquired during a previous training phase, carried out in absence of collisions; such information is then used to compute thresholds from a statistical distribution. In our approach, instead, the monitoring process is handled in parallel, with the only aim of determining a fine, customized value for the sensor sensitivity. Once determined, it is immediately adopted in the collision detection procedure, while starting a further adaptation process to cope with possible slow variations of the robot behavior. In this way, the sensor sensitivity becomes a function of time, properly and continuously refining its value. It must be underlined that there is no proper, dynamic adaptive law, like in the adaptive filtering solution proposed in [14], in the model-based approach in [12] and in the adaptive torque estimation developed in [18], but simply a slow adaptation process introducing small or very small corrections to the sensor sensitivity value, so to keep low the computational burden in real time.

The developed virtual collision sensor is oriented to be used in the industrial context, with the purpose to be both very robust to false detection and characterized by an adjustable sensitivity, so that a collision can be quickly detected, before any damage either of the workspace or of the robot itself might occur; HMI applications are not included as a use case of the proposed virtual sensor. Additional interesting features of the proposed approach are the absence of any initial set-up phase and the generalization of the software implementation.

It is important to underline that the proposed approach, even if fully developed in the COMAU environment, has a general validity, and it can be used and implemented in the control software architecture of the industrial robots of any manufacturer, as well as applied to manipulators devoted to lab activities, provided that the motor currents are on-line measured and some estimate of them is available, possibly computed on the basis of the reference trajectory imposed on the robot.

After a brief overview of the most common model-based collision detection methods in Section 1.1, the collision detection problem is formally stated in Section 2. The working characteristics of the proposed virtual collision sensor are illustrated in Section 3, with details about the model error estimation in Section 3.1 and monitoring of the currents and of their time derivatives in Section 3.2 and Section 3.2.1, respectively. Section 4 is devoted to the automatic learning and adaptation process of the collision sensor sensitivity, whereas Section 5 illustrates the whole implementation structure of the virtual sensor. Experimental results are reported and discussed in Section 6. Section 7 finally draws some conclusions.

### 1.1. Model-Based Approaches for Collision Detection

This section investigates the most common collision detection approaches, using proprioceptive sensors only like the proposed one, but requiring the actual knowledge of the robot dynamic model, in order to show the differences with respect to the developed virtual sensor, as well as to discuss pros and cons.

Two main kinds of approaches belong to the class of model-based collision detection methods: The Residual OBserver (ROB) methods and the Disturbance OBserver (DOB) ones.

In the first technique, proposed in [9,11], the collision detection is performed monitoring the evolution of a residual term, computed by processing the generalized momentum of the robot manipulator through a residual observer. A pro of this method is the avoidance of any acceleration measurement, but the computation of the manipulator inertia matrix (on the basis of the available dynamic model) is required to determine the total energy of the robot. An adaptive version was proposed in [12] to cope with problems related to the model errors, but increasing in this way its complexity. Further ROB approaches were subsequently developed, either aimed at improving the performances, e.g., using dynamic (instead of static) thresholds to monitor the residual term [19,20] or specifically adapted to particular tasks, like in [21,22], where a technique was developed to distinguish the effects of unintended collisions from the forces due to cooperative tasks in HMI applications.

The DOB model-based approach [10,13] is based on the introduction of an observer of the disturbance forces. It was developed directly exploiting the knowledge of the dynamic robot model to estimate all of the disturbance forces acting on the robot, including also the external ones. A threshold is used to monitor the level of external forces and eventually detect the collision. Some solutions were proposed to improve the performances of this method, reducing its sensitivity to model errors. An interesting example was the application of an appropriate band-pass filter to the disturbance torque, so that all of the errors included in the band of the mechanical motion of the robot (that are the main responsible of the overall model error) can be attenuated, as well as the errors at high frequency caused by the noise of the sensors. Using this approach, only the contribution of external forces should be preserved from filtering.

A known problem of model-based approaches is the presence of model errors that degrade the estimate of external forces. In ROB, the effects of model errors are reduced removing the dependency of the residual by the acceleration values; this can be very important because the acceleration values are not measured by a sensor, but they are computed by derivation. A second limitation of the model-based approach is its connection with the particular robot; the requirement of knowing the parameters of the robot implies a customization of the software in the implementation phase to each single manipulator (otherwise, the model errors would increase, and the risk of detecting false collision would grow, as well); so that, even though the overall approach can be defined as general, its software implementation cannot.

The proposed virtual collision sensor tries to overcome these drawbacks, providing an actual general solution, robust to false collision detection, as requested in the industrial scenario.

## 2. Problem Statement and System Characteristics

The goal is the development of a virtual collision sensor, easily applicable to various types of manipulators (i.e., both low-payload and high-payload robots and/or with different kinematic structures), neither requiring specific customizations, nor inserting further, ad hoc sensors beyond the standard ones generally equipping any industrial robot. The virtual collision sensor must contribute to guarantee the mechanical integrity of the robot and the cell and the correct execution of the working process, avoiding false collision alarms that would stop the production cycle. Moreover, it should correctly work without the necessity of a too long warm up phase, before reaching a good level of reliability in detecting an actual collision. In such a case, in fact, any stop of the normal activity of the robot could lead to a loss of accuracy and the need to warm up again.

DC motors are considered as actuators, and the only available real sensors are supposed to be:

- the encoders, mounted on the motor shafts;
- the current sensors, providing the currents absorbed by the motors.

The information provided by the encoders will not be actually employed by the virtual sensor: it would be useful, in fact, only to estimate the joint torques on the basis of the robot dynamic model, which is a solution that has been discarded (as previously discussed) just to avoid any dependence of the collision detection procedure on the characteristics of the specific robot.

**Figure 1:**
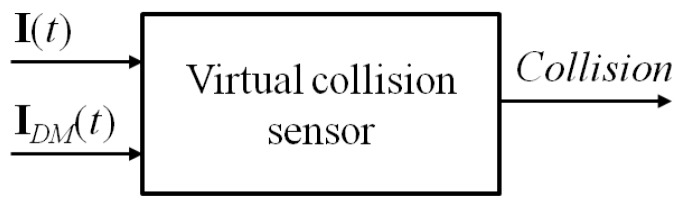
Virtual collision sensor scheme.

The only actual assumption, which the proposed collision detection approach will rely on, is the availability of the estimates of the motor currents, used inside the original robot controller. Such estimates are computed on the basis of an internal dynamic model of the manipulator, whose structure and parameters are not known. This assumption is quite realistic, independent of the particular control scheme adopted. Let $I\left(t\right)=[I_{i}\left(t\right)]$, i=1,...,n, be the vector of the measured currents of the robot motors, where *n* is the number of joints. The typical control scheme of an industrial robot builds such a command current vector as the sum of a feedforward term $I_{ff}\left(t\right)$, pre-computed using the reference trajectory samples of the joint positions, velocities and accelerations, and a feedback control term $I_{r}\left(t\right)$, which is determined by the specific implemented controller (it could be simply the output of a PID-type joint regulator or the result of a more complex control law). The feedforward term is equal to or includes the vector $I_{DM}\left(t\right)=\left[I_{DM,i}\left(t\right)\right]$, i=1,...,n, of the currents estimated by the robot dynamic model. Such a vector is assumed to be available to the virtual collision sensor, together with the measured currents collected in I(t). On the basis of this information only, the virtual sensor will have to continuously update the *n*-dimensional Collision vector (as in the scheme reported in Figure 1), collecting a logical variable for each joint; when at least one of such variables becomes TRUE uppercase? , a collision is detected, and a proper stopping procedure will be immediately applied to the robot.

## 3. The Proposed Collision Detection Virtual Sensor

The virtual collision sensor action is based on the computation of the current residue vector $R\left(t\right)=[R_{i}\left(t\right)]$, i=1,...,n, given by:

(1) $$R\left(t\right)=I\left(t\right)-I_{DM}\left(t\right)$$

where the estimated current vector $I_{DM}\left(t\right)$ is assumed to have been computed by the internal robot dynamic model in absence of any collision, i.e., the complete expression of $I_{DM}\left(t\right)$, if available, would be of the following type:

(2) $$I_{DM}\left(t\right)=K_{I}^{-1}\left(\hat{M}\left(q_{d}\right){\ddot{q}}_{d}+\hat{n}\left(q_{d},{\dot{q}}_{d}\right)\right)$$

where $q_{d}\left(t\right)$, ${\dot{q}}_{d}\left(t\right)$ and ${\ddot{q}}_{d}\left(t\right)$ are the reference joint position, velocity and acceleration vectors, respectively, $\hat{M}\left(\cdot \right)$ is an estimate of the robot inertia matrix, $\hat{n}\left(\cdot \right)$ includes the estimates of the torques due to centrifugal and Coriolis effects, friction and gravity and $K_{I}$ is the diagonal matrix of the conversion coefficients $K_{I,i}$ of the motors (from current to torque).

In the ideal case, i.e., if the internal dynamic model were able to exactly replicate the behavior of the robot, the current residue R(t) would be zero in absence of any collision. In a real case, some model error is always present, also when a good dynamic model is available, so that the current residue is expected to be small on average, but never identically zero. When a collision occurs, the current residue immediately grows, because in this case, the measured motor currents include also the effects of the torques applied to the joints due to the collision forces.

The working mode of the developed virtual collision sensor is based on the comparison of the current residue R(t) with a proper smart threshold, positive-value, vector function S(t) (including a varying threshold $S_{i}\left(t\right)$ for each joint), according to the following collision detection conditions:

(3) $$\left\{\begin{array}{cc} \mathrm{If}\;\;\left|R_{i}\left(t\right)\right|>S_{i}\left(t\right) & \mathrm{then}\;Collision_{i}=TRUE\\ \mathrm{If}\;\;\left|R_{i}\left(t\right)\right|\leq S_{i}\left(t\right) & \mathrm{then}\;Collision_{i}=FALSE \end{array}\right.$$

Too simplistic solutions for the definition of S(t) have to be discarded; for example, a constant threshold value for each joint would unlikely guarantee the required robustness to false collision detection, even if computed on some kind of prior knowledge of the robot behavior (e.g., acquired during a previous, complete work-cycle). An experimental investigation considering various motions for different COMAU manipulators in absence of collisions showed that the current residue values can vary from some tenths of an ampere up to some amperes. This behavior justifies the adoption of a proper smart threshold function in the collision test.

The solution proposed in [15] and here revised is given by the introduction of a threshold varying function, defined as:

(4) $$S\left(t\right)={\hat{m}}_{err}\left(t\right)+{\mathrm{Coll}}_{bias}\left(t\right)$$

the first term, ${\hat{m}}_{err}\left(t\right)$, represents an estimate of the absolute value of the model error in absence of collisions. Such an estimate is determined using the only available information, i.e., the current residue R(t) computed inside the virtual collision sensor as in (1). The second term in (4), ${\mathrm{Coll}}_{bias}\left(t\right)$, represents the sensitivity of the virtual sensor; its entries are positive and are given by the current values corresponding to the minimum collision torque that the virtual sensor should be able to detect on each joint.

The ability of the proposed virtual sensor of detecting collisions, avoiding false alarms, does not depend on the actual quality of the robot dynamic model (which is unknown), but on the capacity of the virtual sensor itself of computing a reliable estimate ${\hat{m}}_{err}\left(t\right)$ of the model error. Considering the adopted expression (4) for the threshold function S(t), the absence of any collision at time *t* is correctly detected on the *i*-th joint by the second condition in (3) if:

(5) $$\Delta m_{err,i}\left(t\right)\leq Coll_{bias,i}\left(t\right)$$

where:

(6) $$\Delta m_{err,i}\left(t\right)=\left|R_{i}\left(t\right)\right|-{\hat{m}}_{err,i}\left(t\right)$$

Inequality (5) shows that small values can be adopted for $Coll_{bias,i}$, i.e., a fine sensitivity of the sensor can be achieved, if $\Delta m_{err,i}\left(t\right)$ is sufficiently small; otherwise, $Coll_{bias,i}$ must be increased to avoid false collision alarms.

In [15], ${\mathrm{Coll}}_{bias}\left(t\right)$ was assumed as constant for all of the joints, simply leaving the user the possibility of changing it, if necessary. In practice, a unique value was adopted for all of the joints and maintained for any robot to which the collision detection procedure was applied. The results were satisfactory, but open to possible improvements, in particular with reference to robustness and speed in detecting a collision. In this paper, the algorithms used in [15] for the computation of ${\hat{m}}_{err}\left(t\right)$ are only slightly modified, since they allowed good results even for the simplistic choice of a constant ${\mathrm{Coll}}_{bias}$ vector, while an automatic learning and adaptation process of the entries of ${\mathrm{Coll}}_{bias}\left(t\right)$ is now proposed, to enhance the robustness of the procedure and the speed in detecting a collision, as well as to cope with possible slow variations of the robot behavior. The model error estimation process is described in SubSection 3.1, while the automatic adaptation of the sensor sensitivity will be developed in Section 4, after having illustrated in SubSection 3.2 the FSM used to monitor the currents behavior.

### 3.1. Model Error Estimation

By the direct analysis of the behavior of the motor currents during any movement of a robot, independently of the specific manipulator and the specific (unknown) internal dynamic model providing $I_{DM}\left(t\right)$, it experimentally results that:

- in the motion phases in which $I_{DM}\left(t\right)$ is almost constant (or it is varying very slowly), the residue R(t) shows a similar behavior, i.e., it is almost constant or slowly varying;
- when $I_{DM}\left(t\right)$ rapidly varies, also the residue does, possibly reaching very high values for some joint.

**Figure 2:**
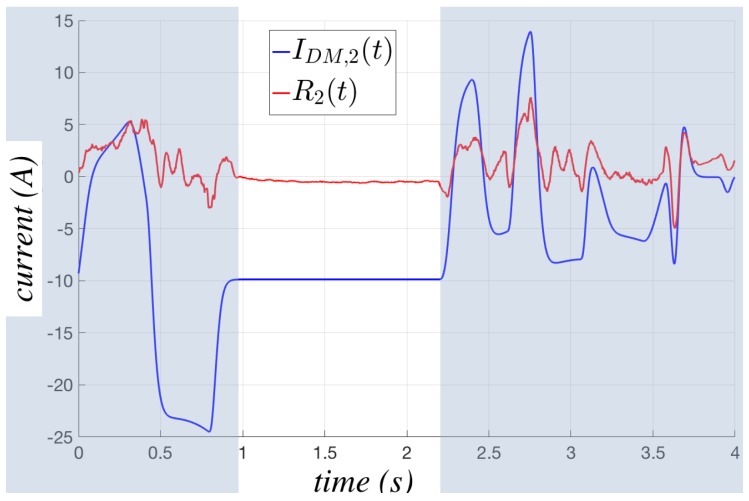
Behavior of the current residue of the second joint of an NJ4 110 in the steady (white background) and unsteady (blue background) states.

The two situations indicate that when the current is almost constant, i.e., when the corresponding torque applied to the joint is almost constant, the dynamic behavior of the system is intrinsically easier to be reconstructed by the internal dynamic model, whatever it is. On the contrary, when the robot is in an acceleration or deceleration phase, i.e., when the currents are rapidly varying, the model error automatically tends to grow, because more complex dynamic effects are acting on the robot, and their estimation is typically and reasonably more difficult. Such observations lead to the opportunity of estimating the model error using different algorithms in the two situations, which will be denoted as steady state and unsteady state, respectively. Figure 2 shows a small portion of a real work cycle carried out by a NJ4 110 robot employed in an automotive production line, highlighting the different behavior of the current residue during the steady state (white background) and the unsteady one (blue background).

During the steady state, the model error for the *i*-th joint can be simply estimated on the basis of an average process, considering the maximum values reached (in absolute value) by the residue during such a steady state time interval, denoted by $T_{ss}$, from the time instant $t_{ss}$, at which the FSM enters the steady state, up to the current time instant *t*. The process starting at time $t_{ss}$ can be expressed as:

(7) $${\bar{ERR}}_{ss,i}\left(t\right)=\frac{1}{N+1}\left(\left|R_{i}\left(t_{ss}\right)\right|+\sum_{\tau \in T_{ss}}{\bar{err}}_{ss,i}\left(\tau \right)\right)$$

where $\left|R_{i}\left(t_{\mathrm{ss}}\right)\right|$ is the last residue value obtained before the transition to the steady state, ${\bar{err}}_{ss,i}\left(\cdot \right)$ is the function containing the maximum values reached by $\left|R_{i}\left(t\right)\right|$ during the steady state until the current time instant *t*, whilst *N* is the number of samples of the function in the same interval. The usage of $\left|R_{i}\left(t_{\mathrm{ss}}\right)\right|$ has been introduced in order to allow a more rapid settling of the values provided by the average process, so as to avoid too small values of ${\bar{ERR}}_{ss,i}\left(t\right)$ at the very beginning of the steady state.

The model error for the *i*-th joint is then computed by sampling ${\bar{ERR}}_{ss,i}\left(t\right)$ with a proper sampling time $T_{s}$, so obtaining a model error defined as:

(8) $${\hat{m}}_{err,i}\left(t\right)=\{\begin{array}{cc} {\bar{ERR}}_{ss,i}\left(t\right) & t=k\;T_{s}\\ {\bar{ERR}}_{ss,i}\left(\left(k-1\right)T_{s}\right) & t\in \left[\left(k-1\right)T_{s},\;k\;T_{s}\right) \end{array}$$

as shown in (8), ${\hat{m}}_{err,i}\left(t\right)$ is actually updated only at time instants $t=k\;T_{s}$. A proper choice of $T_{s}$ is necessary to allow the correct detection of collision. In particular, $T_{s}$ cannot be too small, otherwise it would not be possible to properly distinguish the residue and the model error during the steady state, in which both $I_{DM,i}\left(t\right)$ and $I_{i}\left(t\right)$ change very slowly (and the residue, as well) in absence of collisions. On the contrary, $T_{s}$ cannot be too high, otherwise the actual variation of the residue could not be correctly captured. From a data-driven analysis of the collision timing, $T_{s}$ has been set equal to 0.2 s.

During an unsteady state, two functions, computed during every unsteady state, are combined to estimate the model error for the *i*-th joint as:

(9) $${\hat{m}}_{err,i}\left(t\right)=\delta \;est_{err,i}\left(t\right)+\left(1-\delta \right){\bar{err}}_{us,i}\left(t\right)$$

where ${\bar{err}}_{us,i}\left(t\right)$ is the function containing the maximum values reached by $\left|R_{i}\left(t\right)\right|$ during an unsteady state, $est_{err,i}\left(t\right)$ is computed on a predefined number of residue samples, saved in a buffer, and δ∈[0,1] weighs the contributions of the two terms. In particular, $est_{err,i}\left(t\right)$ is given by:

(10) $$est_{err,i}\left(t\right)={\bar{ERR}}_{us,i}\left(t\right)+3\cdot \sqrt{\frac{1}{N}\sum_{\tau \in T_{us}}{\left({\bar{ERR}}_{us,i}\left(\tau \right)-{\bar{err}}_{us,i}\left(\tau \right)\right)}^{2}}$$

where $T_{us}$ is the time set in which the currents are in the unsteady state, and:

(11) $${\bar{ERR}}_{us,i}\left(t\right)=\frac{1}{N}\sum_{\tau \in T_{us}}{\bar{err}}_{us,i}\left(\tau \right)$$

**Figure 3:**
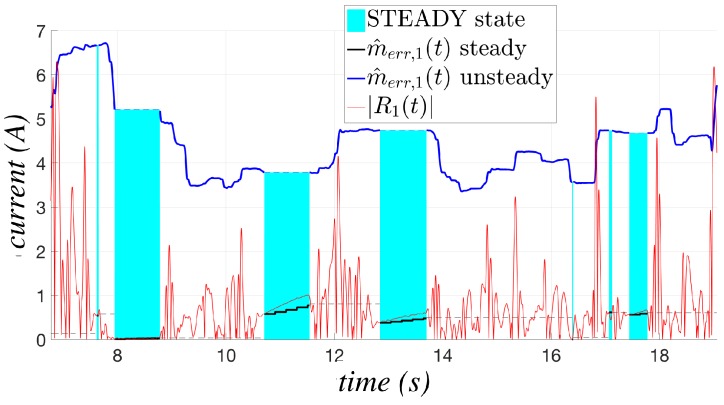
Comparison between ${\hat{m}}_{err,1}\left(t\right)$ computed for the steady state and for unsteady ones.

**Figure 4:**
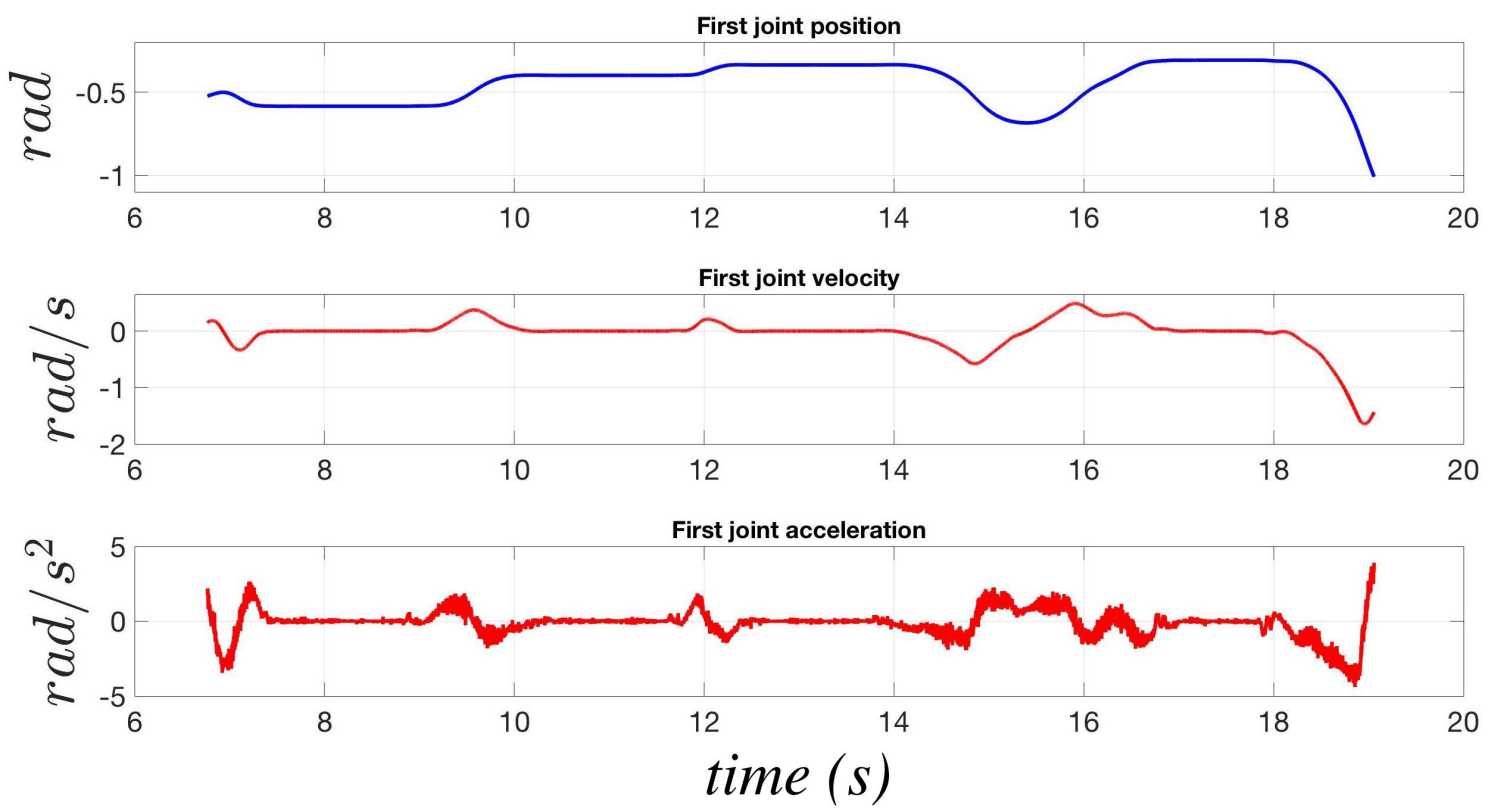
Position, velocity and acceleration of the first joint of the NJ4 170 robot.

Figure 3 shows the behavior of the two model errors for the first joint of the NJ4 170 (whose technical characteristics are available on the COMAU website [23] with an estimated payload of 124.5 kg, while executing the motion reported in Figure 4. The phases in which $I_{DM,1}\left(t\right)$ is in steady state are highlighted by a cyan background. The black line, corresponding to the estimate of the model error in the steady state, is applied only in such cyan phases, whereas the model error in blue is applied the rest of time; such a solution allows one to considerably improve the sensitivity of the algorithm, which can adapt its behavior on the basis of the actual working conditions. The results in Figure 3 have been obtained setting δ=0.5 as in all of the experimental tests reported in Section 6.

**Figure 5:**
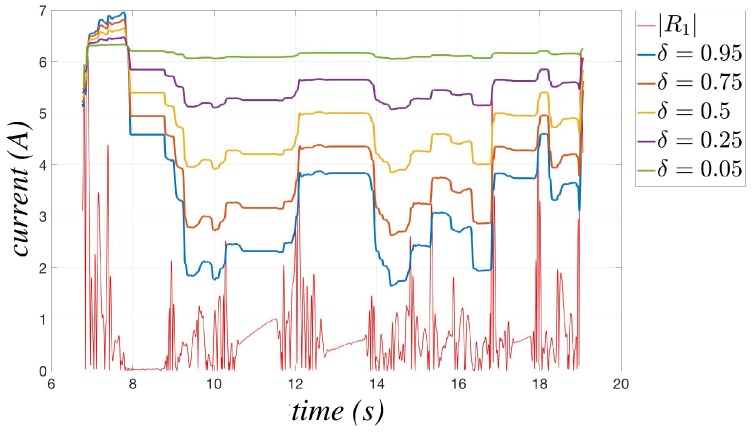
Behavior of the model error ${\hat{m}}_{err,1}\left(t\right)$ in the unsteady states for different values of δ

The change of the parameter δ modifies the behavior of ${\hat{m}}_{err,i}\left(t\right)$ for the unsteady states. When its value is decreased, the model error is more influenced by the maximum value reached during the whole elaboration, so obtaining a procedure more reliable in terms of false collisions, but less rapid to adapt its behavior to the new trend of the residue (e.g., in Figure 5, for δ=0.05, the model error remains close to the maximum reached value). On the contrary, when δ is increased, the model error estimation becomes more reactive, so improving the sensibility of the algorithm (e.g., in Figure 5, for δ=0.95, the model error remains close to the residual values), but simultaneously increasing the risk of false collision alarms. The adoption of a proper trade-off value is then recommended.

The transition between the steady state estimation and the unsteady one is performed without any particular management; despite this choice leading to discontinuities in the computation of the threshold (4), such an approach does not cause any functional anomaly in practice, so that it can be adopted to keep the computational burden low in the real-time implementation of the virtual sensor.

**Remark** **1.** *When the payload declaration is wrong (or the payload is changed without updating the relative information in the control scheme and, hence, in the adopted dynamic model), a poor current estimate* $I_{DM}$ *could be provided, leading to the risk of possible failures in the collision detection. When the error in the payload declaration is small (e.g., in the case of an imprecise auto-determination of the payload), the algorithm can still correctly work, thanks to the automatic convergence of the estimated model error to higher values. Instead, when the user payload declaration is totally wrong, the virtual sensor could provide a false collision output, which actually corresponds to the detection of the anomalous situation generated by the wrong payload declaration, like in the case of a work-piece remaining accidentally attached to the gripper, whose effects are similar to those of a collision.*

In the self-identification procedure of the payload used by COMAU, the estimate of the payload parameters is sufficiently accurate to avoid false collisions with the proposed virtual sensor; on the contrary, when the procedure is modified by the user (e.g., in order to reduce the stroke of the joints), the quality of the achieved estimate generally worsens, leading to a possible false collision detection (as in the case of explicit wrong declaration of the payload).

*It is important to note that* $I_{DM}$ *is used in the robot control system, so that payload declaration errors could worsen the motion accuracy, especially in the acceleration and deceleration phases with possible low frequency vibrations, which could contribute to the detection of false collisions.*

### 3.2. Monitoring of the Currents’ Behavior through an FSM

The trends of the currents I(t) and $I_{DM}\left(t\right)$, i.e., the inputs of the virtual sensor, are monitored to distinguish:

- steady and unsteady states, to apply the most suitable model error estimation algorithm;
- unsafe and safe states, i.e., situations in which collisions might actually occur or not, so to perform the collision detection test only in the unsafe ones, thus enhancing the efficiency of the virtual sensor implementation.

The monitoring action is performed implementing a five-state FSM for each joint, after having applied a proper filtering action to both I(t) and $I_{DM}\left(t\right)$. Such a filtering action, which is mandatory for the measured current, is applied as-it-is to the estimated one, as well, so to avoid any time delay between them. A low-pass filter with a 10–20-Hz bandwidth can represent a satisfactory solution in general.

**Figure 6:**
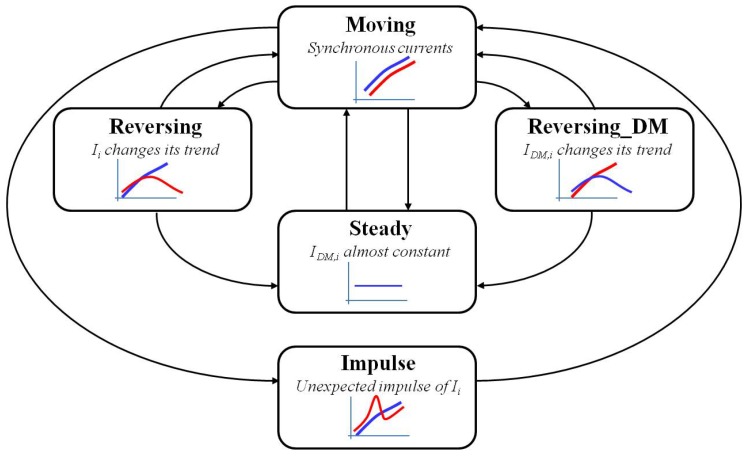
FSM scheme with the sketch of the currents behavior in the various states ($I_{i}$ in red and $I_{DM,i}$ in blue).

**Figure 7:**
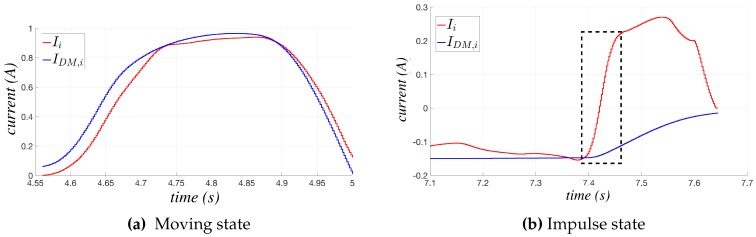
Examples of the behavior of $I_{i}\left(t\right)$ and $I_{DM,i}\left(t\right)$ during the moving and the impulse states.

The five states of the FSM of the *i*-th joint, which is reported in Figure 6 with a sketch of the currents behavior in the various states ($I_{i}\left(t\right)$ in red and $I_{DM,i}\left(t\right)$ in blue), are:

- Steady state, in which the estimated current $I_{DM,i}\left(t\right)$ is almost constant or very slowly varying (as in the phases having white background in Figure 2); this state is recognized by computing the first and the second order time derivatives of $I_{DM,i}\left(t\right)$, which must tend both to zero. It is worth noting that $I_{DM,i}\left(t\right)$ is not affected by noise, since it is provided by the internal robot dynamic model on the basis of the reference joint trajectory, so that the time derivative computation can be made without any numerical problem.
- Moving state, in which the current values of $I_{i}\left(t\right)$ and $I_{DM,i}\left(t\right)$ vary rapidly, but remaining synchronous, as in Figure 7a; this state is distinguished by the previous one monitoring the time derivative of both $I_{i}\left(t\right)$ and $I_{DM,i}\left(t\right)$, denoted as $dp\_I_{i}\left(t\right)$ and $dp\_I_{DM,i}\left(t\right)$, respectively: when they start to increase in absolute value, the steady state is abandoned, and the FSM switches to the moving state. The synchronicity of the currents is detected by comparing $dp\_I_{i}\left(t\right)$ and $dp\_I_{DM,i}\left(t\right)$, as detailed in Subsection 3.2.1.
- Reversing and reversing_DM states, in which only one of the two currents, $I_{i}\left(t\right)$ or $I_{DM,i}\left(t\right)$, changes its trend, i.e., the sign of its time derivative changes; two states of reversing type are used to distinguish the two possible cases, i.e., which of the two currents is changing its trend, as sketched in Figure 6.
- Impulse state, in which sudden impulses of $I_{i}\left(t\right)$, which are not present in $I_{DM,i}\left(t\right)$ occur (see Figure 7b); this state is recognized by monitoring the error between the time derivatives of the currents.

The two reversing states and the impulse one, in which the measured and the estimated currents are not accordingly varying, are surely unsafe states, in which the anomalous currents behavior may be due to a collision. The moving and the steady states should correspond to the standard working conditions of the robot, in which acceleration/deceleration phases alternate with the constant velocity ones, but only the moving state can be surely considered as safe; in fact, the steady one is recognized on the basis of the estimated current behavior only, so that for the sake of robustness, it is convenient to handle it as a potentially unsafe state.

On the basis of such a distinction between safe and unsafe states, the virtual sensor will avoid performing the collision test based on (3) when the FSM is in the moving state, so to improve its working efficiency; on the contrary, the test will be executed in any other state.

#### 3.2.1. Monitoring of the Currents’ Time Derivatives

**Figure 8:**
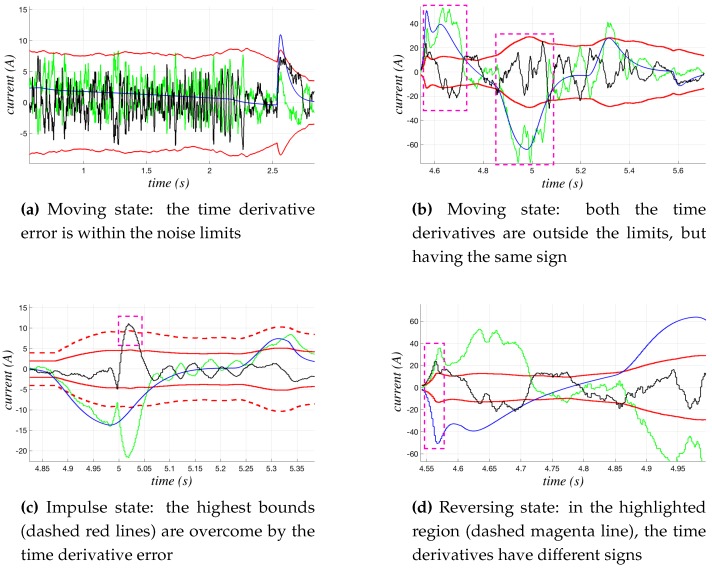
Examples of the behavior of $dp\_I_{i}\left(t\right)$ and $dp\_I_{DM,i}\left(t\right)$ in the moving, reversing and impulse states. The solid red lines indicate the $Th_{max}$ and $Th_{min}$ bounds; the green line and the blue one represent $dp\_I_{i}\left(t\right)$ and $I_{DM,i}\left(t\right)$, respectively, and the solid black line their difference, computed as $dp\_I_{i}\left(t\right)$ - $dp\_I_{DM,i}\left(t\right)$.

In all of the states, but the steady one, the time derivatives of both the measured and the estimated currents have to be computed and monitored. The noise that inevitably affects the measured current makes the pure numerical computation of its derivative unsuitable for our purposes. The insertion of a filter with a narrow or very narrow band has to be avoided, because it could lead to unacceptable delays in detecting changes of the current signal trend and, hence, in detecting a possible collision. The adopted solution is based on the dynamical estimation of the noise affecting $dp\_I_{i}\left(t\right)$, via the statistical computation of the error between the time derivatives of the two currents (since $I_{DM,i}\left(t\right)$ is not affected by noise, also its time derivative is not). Such a result is used to define an upper bound $Th_{max}$ and a lower bound $Th_{min}$ of $dp\_I_{i}\left(t\right)$, as shown in Figure 8, where the limits are indicated by solid red lines.

In particular, the FSM is in the moving state if one of the two following situation occurs: (i) the difference between $dp\_I_{i}\left(t\right)$ and $dp\_I_{DM,i}\left(t\right)$ is within the noise limits (see Figure 8a), (ii) $dp\_I_{i}\left(t\right)$ and $dp\_I_{DM,i}\left(t\right)$ are both over the limits, but they have the same sign (see Figure 8b). However, when the first case occurs, if a very rapid impulse of the current values brings the error to overcome a further much higher bound (dashed red lines in Figure 8c), the FSM changes its state into impulse. As in the second case of the moving state, for the reversing and the reversing_DM states, the error between $dp\_I_{i}\left(t\right)$ and $dp\_I_{DM,i}\left(t\right)$ and their signs are both monitored, but in this case, the change of the FSM occurs when the error is over the limits and the signs of the time derivatives are different (see Figure 8d).

**Remark** **2.** *The insertion of the filtering action on the measured and estimated currents and the estimation of the model error through average processes would determine an initial, transient phase in the computation of* S(t)*, in which the collision detection could be not fully reliable. This is not a problem in practice, since the duration of such a time interval is generally smaller than the waiting phase that is usually set by the robot constructors after the launch of the “drive-on” state, in which the motors are active and the manipulator is ready to perform the assigned task. In the COMAU case, the duration of this phase is of some ms; such a time interval is more than sufficient to achieve a reliable value of the threshold function, so that the virtual collision sensor will be properly working also at the beginning of the robot motion.*

## 4. Automatic Learning and Adaptation of the Sensor Sensitivity to Collisions

The adoption of a constant vector ${\mathrm{Coll}}_{bias}$ in the definition (4) of the threshold function has allowed satisfactory results for a wide class of manipulators, employed in different robotic applications, simply keeping the same values (heuristically determined) in all of the implementations; some results are reported and discussed in Section 6. Despite this, significant differences have been noted with reference to the actual ability of all of the joints of detecting a collision and/or to the speed in detecting it. The adoption of a unique, constant ${\mathrm{Coll}}_{bias}$ vector can result in quite different levels of sensor sensibility with respect to the specific behavior of each robot, with no possibility of taking into account possible slow variations in the robot behavior as time goes by.

An automatic learning and adaptation process of the sensor sensitivity is now proposed to cope with such problems, under the assumption that the whole motion process of the robot is cyclic, as in any industrial application. The goal is to determine the “best” ${\mathrm{Coll}}_{bias}$ term for the specific robot application through a learning phase and to subsequently apply it enabling a slow adaptation phase, in which further small variations of the robot behavior are automatically taken into account. In this context, the learning process is aimed at automatically finding a customized value for the ${\mathrm{Coll}}_{bias}$ term for the specific installation of the virtual sensor, while the subsequent adaptation process introduces small or very small corrections to such a term, in order to avoid any false collision detection caused by slow changes of the residue values, e.g., due to temperature variations.

**Figure 9:**
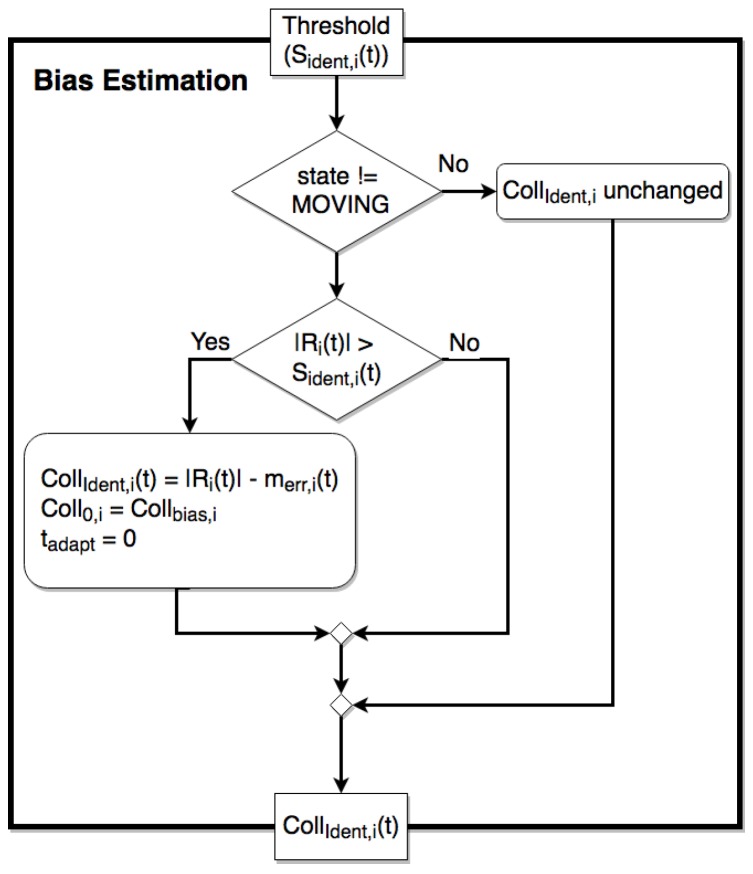
Activity diagram of the bias estimation block for the *i*-th joint.

In the proposed learning and adaptation process, an initial, constant ${\mathrm{Coll}}_{bias_{0}}$ vector is assumed to be available (somehow heuristically determined) and employed as ${\mathrm{Coll}}_{bias}$ in the threshold function S(t) used in the collision test, if the user does not request to adapt it. The entries of such a vector are generally sufficiently high to limit/avoid the risk of false collision detection during the standard, correct execution of the robot moving cycle. A learning bias estimation block is introduced (and kept always active), which executes a parallel collision test, still defined as in (3), but adopting a different threshold function, denoted as $S_{Ident}\left(t\right)$, whose *i*-th entry is defined as:

(12) $$S_{Ident,i}\left(t\right)={\hat{m}}_{err,i}\left(t\right)+Coll_{Ident,i}\left(t\right)$$

in which ${\hat{m}}_{err,i}\left(t\right)$ is still computed as in (8) and (9) in the steady and unsteady states, respectively, while $Coll_{Ident,i}\left(t\right)$ is going to be updated as in the activity diagram reported in Figure 9, starting from $Coll_{Ident,i}\left(0\right)=0$. This initial choice intentionally leads to a virtual (false) collision detection by the bias estimation block, when the collision condition $\left|R_{i}\left(t\right)\right|>S_{Ident,i}\left(t\right)$ holds for the *i*-th joint. No collision actually occurs, but such a condition is used to update $Coll_{Ident,i}\left(t\right)$ imposing:

(13) $$Coll_{Ident,i}\left(t\right)=\left|R_{i}\left(t\right)\right|-{\hat{m}}_{err,i}\left(t\right)$$

i.e., equal to the minimum value, which would allow one to avoid a false collision detection, if used in the main collision test. The minimum duration of the learning process is set according to the characteristics and duration of the whole motion that the robot cyclically repeats (e.g., a pick-and-place cycle). At least an entire cycle must be monitored during the learning phase to obtain a reliable estimate of the minimum $Coll_{Ident,i}\left(t\right)$ (denoted as ${\bar{Coll}}_{Ident,i}^{\left(0\right)}$) that should be adopted, but a longer learning time can be imposed for the sake of robustness; for example, in the experimental tests reported and discussed in Section 6, a learning time of three cycles is considered.

Further actions are performed by the bias estimation block, when a virtual (false) collision is detected, to initialize a possible subsequent adaptation process, which actually starts only if and when the user requests it. It must be underlined that even if the bias estimation block is always active, and hence $Coll_{Ident,i}$ is continuously updated, no change is introduced in (4) in the main collision test until the user’s request. Such a request determines the immediate application of the new bias value (as soon as the minimum learning time has passed), and the start of a slow adaptation of $Coll_{bias,i}$ by defining:

(14) $$Coll_{bias,i}\left(t_{adapt}\right)={\bar{Coll}}_{Ident,i}^{\left(k\right)}+e^{\left(-t_{adapt}/\tau_{a}\right)}\left(Coll_{0,i}-{\bar{Coll}}_{Ident,i}^{\left(k\right)}\right)$$

where $t_{adapt}$ is a time variable that is set to zero by the bias estimation block each time it detects a virtual (false) collision, ${\bar{Coll}}_{Ident,i}^{\left(k\right)}$ indicates the value of $Coll_{ident,i}$ updated for the *k*-th time by such a block after the start of the adaptation process (k=0 corresponds to the value that directly substitutes the original $Coll_{bias_{0},i}$) and $\tau_{a}$ is the time constant of the adaptation process. $\tau_{a}$ must be much greater than the typical values of collision detection times, so to avoid a too rapid increase of the bias term that could prevent the correct detection of a real collision; since the collision detecting times are expected to be of the order of some tens of ms, $\tau_{a}$ must be chosen so to have a rise time of $Coll_{bias,i}$ of some minutes or tens of minutes. The $Coll_{0,i}$ parameter in (14) is used to force the application of the new bias term at the end of the learning phase and to define the initial condition of any further adaptation process. In particular, the immediate application (after the user’s request) of the first bias term ${\bar{Coll}}_{Ident,i}^{\left(0\right)}$, provided at the end of the learning phase, is simply achieved by imposing:

(15) $$Coll_{0,i}={\bar{Coll}}_{Ident,i}^{\left(0\right)}$$

this implies that the adaptation function (14) will actually change the $Coll_{bias,i}$ value only when the bias estimation block provides a new, updated estimate ${\bar{Coll}}_{Ident,i}^{\left(k\right)}$, with k>0. Each time this happens, this new value is automatically used in (14), while the bias estimation block imposes:

(16) $$\begin{array}{c} Coll_{0,i}=Coll_{bias,i}\\ t_{adapt}=0 \end{array}$$

as indicated in the activity diagram reported in Figure 9. These assignments make the adaptation process (14) restart from the current $Coll_{bias,i}$ value and let it tend to the new ${\bar{Coll}}_{Ident,i}^{\left(k\right)}$. Such a value will be actually, slowly reached, according to the settling time imposed by $\tau_{a}$, only if in the meantime, no further updated value ${\bar{Coll}}_{Ident,i}^{\left(k\right)}$ is provided by the bias estimation block; otherwise, the adaptation process will restart again, imposing the convergence of $Coll_{bias,i}$ to such a new value.

**Remark** **3.** *If the user never asks for the adaptation of the* ${\mathrm{Coll}}_{bias}$ *term,* $t_{adapt}$ *remains always equal to zero, and the original* ${\mathrm{Coll}}_{bias0}$ *vector is indefinitely maintained in the threshold function (4) used in the collision test (3).*

**Figure 10:**
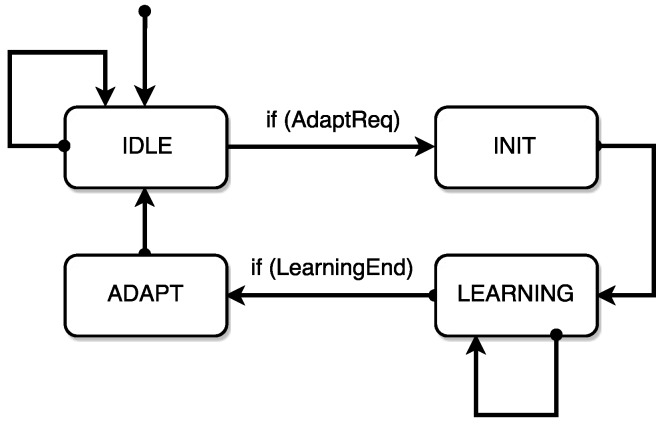
FSM for the sensor sensitivity adaptation.

An FSM included in the virtual collision sensor handles the entire learning and adaptation process, guaranteeing in particular that the adaptive expression (14) of $Coll_{bias,i}\left(t\right)$ is actually used in the collision test only if a sufficient learning time has passed (corresponding to one process cycle or more, according to the user preferences, as previously discussed). This condition is ensured simply maintaining $t_{adapt}=0$ until the established learning time has passed and a first reliable ${\bar{Coll}}_{Ident,i}^{\left(0\right)}$ value has been computed. The FSM (sketched in Figure 10) uses four states to manage the learning and adaptation process through three main services:

- Init, corresponding to the user’s request of a new learning and adaptation process of ${\mathrm{Coll}}_{bias}$. It sets to zero all the entries of ${\mathrm{Coll}}_{ident}$ and the time variable $t_{adapt}$; such a variable will remain locked to zero until the beginning of the adaptation phase, enabled by the subsequent adapt service.
- Set: It allows the direct application of the new ${\bar{\mathrm{Coll}}}_{Ident}^{\left(0\right)}$ vector estimated during the learning phase, from which the slow adaptation process will start.
- Adapt: it lets the adaptation process (14) start, unlocking the time variable $t_{adapt}$, so that the vector ${\mathrm{Coll}}_{bias}$ in (4) becomes a slow function of time.

The sequence of operations performed in the four states of the FSM can then be summarized as follows:

1. **IDLE** : The FSM remains in the IDLE state until an adaptation request is received. The values of the ${\mathrm{Coll}}_{Ident}$ vector are continuously updated by the bias estimation block, but their values do not affect ${\mathrm{Coll}}_{bias}$ and the threshold function (4) actually used in the collision test. The user can send a request (AdaptReq) using a specific instruction to be inserted in the user program.
2. **INIT**: The FSM launches the Init service, so that the bias estimation process restarts from ${\mathrm{Coll}}_{Ident}=0$ (any previous value of ${\mathrm{Coll}}_{Ident}$ is discarded).
3. **LEARNING**: The FSM remains in the LEARNING state until the imposed learning time has passed and a reliable ${\bar{\mathrm{Coll}}}_{Ident}^{\left(0\right)}$ vector has been determined by the bias estimation block. When such a waiting phase is over (LearningEnd), while leaving the LEARNING state the set service directly applies the new ${\bar{\mathrm{Coll}}}_{Ident}^{\left(0\right)}$ vector.
4. **ADAPT**: The FSM launches the adapt service and then comes back to IDLE, leaving the bias estimation block and the adaptation law (14) both active.

**Figure 11:**
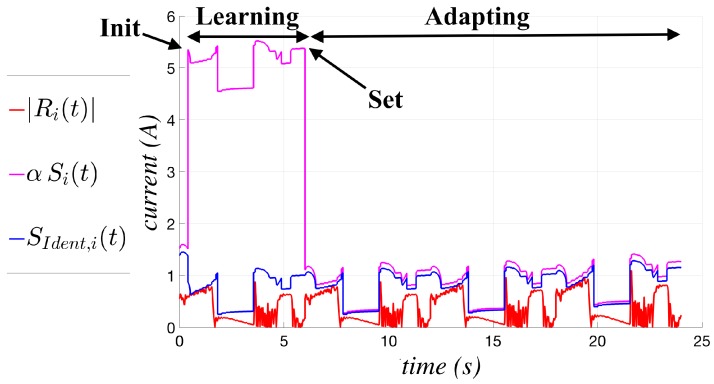
Example of a cycle involving init, learning, set and adapting phases.

A complete cycle involving the initialization (init), learning, set and adapting phases is shown in Figure 11; it highlights the great difference in behavior of the threshold function before and after the set instant. The figure compares the current residue (in absolute value) $R_{i}\left(t\right)$, the identified threshold function $S_{Ident,i}\left(t\right)$ and the threshold function actually applied in the collision test, defined as $\alpha S_{i}\left(t\right)$, where the $S_{i}\left(t\right)$ function given in (4) is multiplied by a factor α, slightly greater than one, to avoid possible problems in the practical implementation, as discussed in the next section.

## 5. Whole Structure of the Virtual Collision Sensor

**Figure 12:**
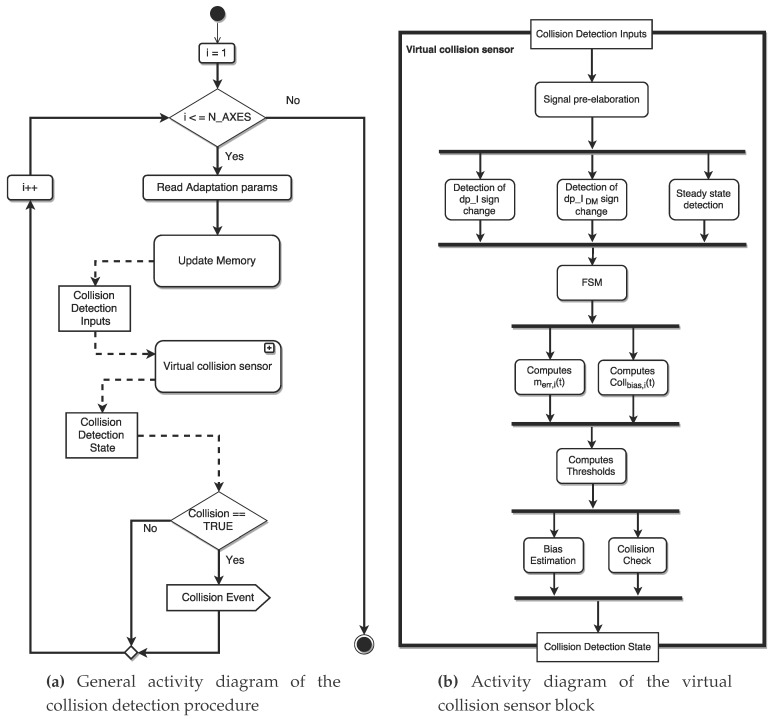
Overall activity diagrams.

The virtual collision sensor is not aware of the robotic structure of the whole system, so that it simply works checking the current values of each joint one by one. The global virtual sensor is then composed by a cycle in which each joint is tested by the virtual collision sensor block; when a collision occurs a collision event is raised, so that a properly post-collision handling can be carried out. As shown in Figure 12a, the call of the virtual collision sensor block is preceded by an initialization phase, in which the parameters related to the activation and the timing of the adaptation phase are read and used for the subsequent update of the memory (update memory block). During such a phase, the object called collision detection state, containing the state of the algorithm, is used together with the new input values (e.g., current values and adaptation parameters) in order to define the inputs for the collision detection procedure (i.e., the object called collision detection inputs).

The flowchart representing the virtual collision sensor block was already presented in [15], but here, some modifications are introduced in order to implement the learning and adaptation of the sensor sensitivity. The workflow of the new virtual collision sensor block (Figure 12b) is presented using the graphical representation provided by the activity diagrams, which allows defining additional characteristics like the parameters involved in the activities and the sets of activities that can be executed in parallel (fork/joint statement).

The first part of the activity flow (i.e., from the starting block to the FSM) is not changed from a conceptual point of view; the input values are pre-elaborated (e.g., applying a filtering action on the input signals), and the first and second order time derivatives of $I_{i}$ and $I_{DM,i}$ are computed. The time derivatives are then used by the subsequent three blocks (which can be executed in parallel) to evaluate if $I_{i}$ and $I_{DM,i}$ change their trend and to detect when $I_{DM,i}$ enters in a steady condition. The FSM works as shown in Figure 6, monitoring the behavior of the current signals.

**Figure 13:**
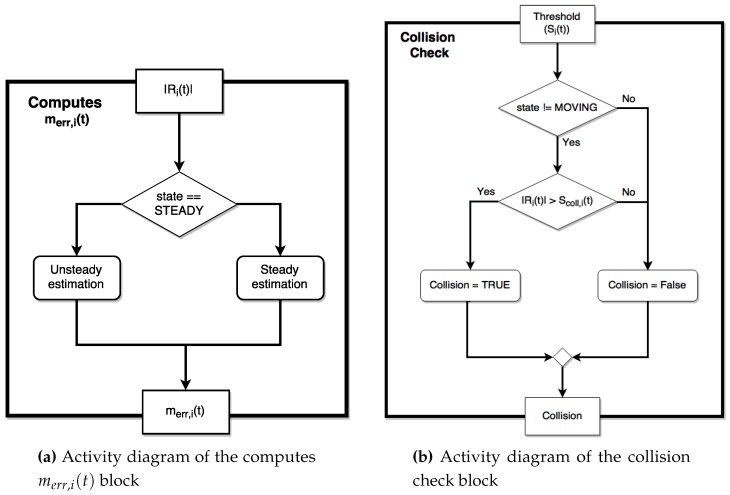
Activity diagrams of the blocks computing the terms of the threshold function.

The rest of the activity diagram has been slightly changed to introduce the new features; in particular, a specific block (called computes $Coll_{bias}$) computing $Coll_{bias,i}\left(t\right)$ using (14) is introduced; such an activity is performed in parallel execution with respect to the computation of the model errors (carried out by the so-called computes ${\hat{m}}_{err}\left(t\right)$ block, reported in Figure 13a). The computes thresholds block performs the computation of the two thresholds S(t) and $S_{Ident}\left(t\right)$, using respectively Equations (4) and (12).

The last two activities are performed in parallel; the first one computes $Coll_{Ident,i}$ as shown in the activity diagram in Figure 9, whereas the second one carries out the collision test (collision check block; see Figure 13b) with small differences with respect to the basic version proposed in [15]. The adaptation phase is based on a parallel updating of $Coll_{Ident,i}\left(t\right)$ without stopping the robot.

**Figure 14:**
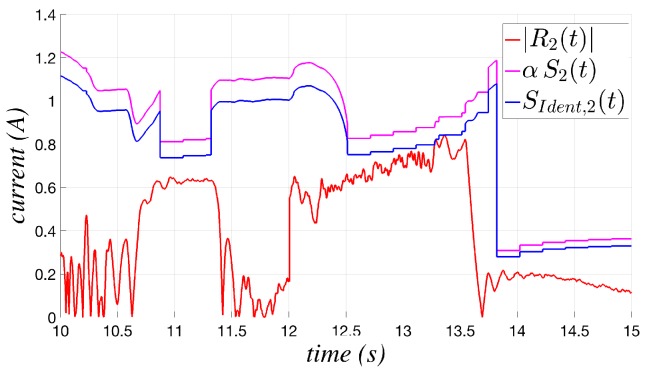
Behavior of threshold functions and current residue during the adaptation phase for the second joint of an NS12 manipulator.

It must be noted that the procedure properly works only if $S_{i}\left(t\right)$ is always greater then $S_{Ident,i}\left(t\right)$, in particular after the end of the learning phase when the subsequent set action is performed; in fact, if such a condition does not hold, whenever the bias estimation block detects a (false) collision, the collision check block would detect it, as well, because in practice, they would perform the same test with the same threshold. In order to avoid this kind of problem, the threshold really applied in the collision test (3) is slightly increased (see the example reported in Figure 14) substituting $S_{i}\left(t\right)$ with:

(17) $$S_{coll,i}\left(t\right)=\alpha S_{i}\left(t\right)$$

where α is slightly greater then one, just to let the threshold function used in the collision check block be always different from the one used in the bias estimation block.

**Remark** **4.** *The usage of the coefficient α does not lead the procedure to become insensitive to real collisions; in fact, after the application of the new bias through the set service, the threshold function decreases drastically with respect to its initial value, so that the α coefficient cannot produce in practice an increase of the threshold function sufficient to let it reach values greater then the initial ones (see Figure 11). In the worst case in which* ${\mathrm{Coll}}_{Ident}$ *is equal to* ${\mathrm{Coll}}_{bias,0}$*, the system would have a worsening of its sensibility of about* (α−1)*% with respect to the basic version.*

## 6. Experimental Results

Experimental tests are carried out in order to compare the performances of the basic version of the procedure proposed in [15] with those of the complete virtual sensor, including the sensitivity learning and adaptation process. The experiments are performed on a NS 12 robot by COMAU (Grugliasco, Italy) by imposing real collisions in some predefined positions of the work-space. The movements are defined using the programming language (i.e., the PDL2) of the COMAU control system (called C5G), through which a cyclical program repeating several movements has been created. The collisions tests are carried out for different types of movement, i.e., when the robot is moving linearly in a plane parallel to the floor (left → right) and when the robot is moving linearly along a line perpendicular to the floor (top → bottom). For both cases, the collisions are imposed in different points of the workspace by placing an obstacle (a cardboard box of about 15 kg) along the line of movement of the robot just during the motion. In order to highlight the behavior in different conditions, some of the collision points are chosen in the central part of the workspace, i.e., near the robot base (denoted as NR in Table 1 and Table 2), whereas some others are taken close to the frontier of the workspace (EOS in Table 1 and Table 2).

For each point of collision, two tests are performed: (i) using the basic version of the algorithm proposed in [15]; (ii) using the adaptive virtual sensor developed here; in the case of the basic version, the collision detection is enabled with the standard thresholds, whereas for the adaptive virtual sensor, an initial learning phase of three cycles is performed before the collision. The obtained results show a very large decrease of the time required to detect the collision when the adaptive version is used, in particular, the detection time of the basic version can be reduced between 56% and 87% (see Table 1). A second important improvement is related to the number of axes able to detect the collision; as shown in Table 2 for this particular experiment, in which the collision with a cardboard box could be difficult to detect because of its low stiffness, the basic algorithm is able to detect it with no more then two axes, whereas the adaptive version detects the collision with almost all joints (and in some cases, just with all of them), thus enhancing the robustness of the collision detection process.

**Figure 15:**
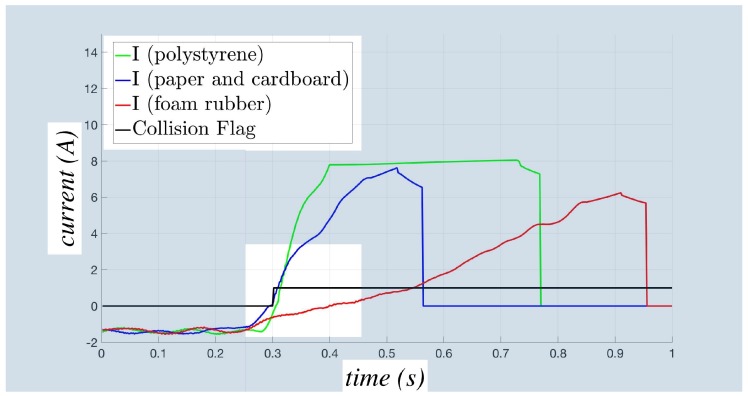
Behavior of the current *I* during the collisions for three different materials.

A further set of experiments is carried out in order to show the behavior of the adaptive virtual sensor in the case of collisions with different materials. The tests are performed on a COMAU NS 12 imposing a vertical motion to provoke a collision with a stack of elements of the following materials: (i) paper and cardboard, (ii) polystyrene and (iii) foam rubber. The detection times of the tests are summarized in Table 3, where it can be seen that the stack of foam rubber requires a longer time because of its low stiffness, whereas polystyrene and paper show similar detection times. Such a result could be attributed to the similar stiffness characteristics of the surfaces of the last two materials, as confirmed also by the current behavior that is reported in Figure 15 for the sixth joint (which is the first one detecting the collision) in the three tests; to facilitate the analysis of the results, the three different detection times have been made coincident. As can be seen in the white background region of the figure, a more rapid increase of the current in the paper/cardboard and in the polystyrene cases allows a faster collision detection than in the foam rubber one. The robustness of the collision detection process is confirmed for all of the materials: as shown in Table 4, only the first joint in the foam rubber case fails to detect the collision, whereas all of the joints detect it with the other two materials. It is worth noticing that the first joint is not directly involved in the motion causing the collision, so that the obtained results prove a high robustness of the virtual collision sensor.

The basic version of the collision detection procedure was gradually applied by COMAU during 2016 to factories and production lines, starting with the Fiat Chrysler Automobiles (FCA) plant of Cassino (Southern Italy), which is mainly composed of high-payload robots involved in handling applications. The procedure was then inserted in further production lines, e.g., in TOFAS (Turkey) in the last supplying of 30 robots, in the Pirelli plant, where after a first test involving just a few robots, the procedure is going to be extended to the rest of the robots pool, and in the FCA plant in Cordoba (Argentina), where it has been installed on about 160 robots. The new adaptive virtual collision sensor here proposed is going to be soon tested in a real industrial context, exploiting the suitability of its characteristics to the cyclical nature of the industrial applications.

## 7. Conclusions

In this paper, an enhanced version of the collision detection algorithm presented in [15] has been proposed to achieve a complete virtual collision sensor. The virtual sensor includes a new feature, activated by the user, that allows one to estimate the most suitable collision sensor sensitivity for the specific task performed by the robot; moreover, a slow adaptation phase is imposed after its application, in order to avoid false collisions due to very slow changes of the motor currents’ behavior. The adaptive version is applicable only in the case of cyclical motions (typical of industrial applications), because it requires an initial learning phase during which the bias term of the threshold function is estimated. The new procedure shows very good improvements with respect to the basic one in terms of both sensibility and robustness, so that it could be used for high precision contact-tasks, as well, if combined with a proper post-collision reaction, e.g, by using an impedance control law; such an issue will be investigated in future works.

## Figures and Tables

**Table 1:** Comparison between the collision Detection Times (DT) of the basic version and the adaptive one.

Performed Tests	DT Adapt (s)	DT Basic (s)	Average Reduction %
top → bottom EOS	0.026	0.106	75.5
top → bottom NR	0.024	0.186	87.1
left → right EOS	0.010	0.044	77.3
left → right NR	0.010	0.046	78.3

**Table 2:** Comparison of the number of axes that are able to detect the collision using the basic collision detection procedure and the proposed virtual sensor, including the learning and adaptive functionalities.

		Ax					
	Performed Tests	1	2	3	4	5	6
Basic	top → bottom EOS	_	•	_	_	_	_
	top → bottom NR	_	_	_	_	•	•
	left → right EOS	•	_	_	_	_	_
	left → right NR	•	_	_	•	_	_
Adaptive	top → bottom EOS	_	•	•	•	•	•
	top → bottom NR	_	•	•	_	•	•
	left → right EOS	•	_	•	•	•	•
	left → right NR	•	•	•	•	•	•

**Table 3:** Collision Detection Times (DT) for the three different materials using the adaptive virtual sensor.

Performed Tests	DT Adapt (s)
paper and cardboard	0.036
polystyrene	0.022
foam rubber	0.076

**Table 4:** Axes able to detect the collision for the three different materials using the adaptive virtual sensor.

		Ax					
	Performed Tests	1	2	3	4	5	6
Adaptive	paper and cardboard	•	•	•	•	•	•
	polystyrene	•	•	•	•	•	•
	foam rubber	_	•	•	•	•	•

## Abbreviations

The following abbreviations are used in this manuscript:

HMI	Human-Machine Interaction
LWR	Light Weight Robot
VST	Variable Stiffness Transmission
ROB	Residual OBserver
DOB	Disturbance OBserver
FSM	Finite State Machine
